# Maintenance Use of Antidepressants in Dutch General Practice: Non-Guideline Concordant

**DOI:** 10.1371/journal.pone.0097463

**Published:** 2014-05-23

**Authors:** Ellen Piek, Boudewijn J. Kollen, Klaas van der Meer, Brenda W. J. H. Penninx, Willem A. Nolen

**Affiliations:** 1 Department of General Practice, University Medical Center Groningen, University of Groningen, Groningen, The Netherlands; 2 Department of Psychiatry, University Medical Center Groningen, University of Groningen, Groningen, The Netherlands; 3 Department of Psychiatry, Leiden University Medical Center, Leiden, The Netherlands; 4 Department of Psychiatry, EMGO Institute for Health and Care Research, VU University Medical Center, Amsterdam, The Netherlands; Universidad de Valladolid, Spain

## Abstract

**Background:**

There is hardly evidence on maintenance treatment with antidepressants in primary care. Nevertheless, depression guidelines recommend maintenance treatment i.e. treatment to prevent recurrences, in patients with high risk of recurrence, and many patients use maintenance treatment with antidepressants. This study explores the characteristics of patients on maintenance treatment with antidepressants in general practice, and compares these characteristics with guideline recommendations for maintenance treatment.

**Methods:**

We used data (baseline, two-year and four-year follow-up) of primary care respondents with remitted depressive disorder (≥6 months) from the Netherlands Study of Depression and Anxiety (n = 776). Maintenance treatment was defined as the use of an antidepressant for ≥12 months. Multilevel logistic regression was used to describe the association between sociodemographic, clinical and care characteristics and use of maintenance treatment with antidepressants.

**Results:**

Older patients, patients with a lower education, those using benzodiazepines or receiving psychological/psychiatric care and patients with a concurrent history of a dysthymic or anxiety disorder more often received maintenance treatment with antidepressants.

**Limitations:**

Measurements were not made at the start of an episode, but at predetermined points in time. Diagnoses were based on interview (CIDI) data and could therefore in some cases have been different from the GP diagnosis.

**Conclusions:**

Since patients with chronic or recurrent depression do not use maintenance treatment with antidepressants more often, characteristics of patients on maintenance treatment do not fully correspond with guideline recommendations. However, patients on maintenance treatment appear to be those with more severe disorder and/or more comorbidity.

## Introduction

Depression is a common condition that has a chronic or recurrent course in a significant proportion of cases [1]. Most patients are treated in primary care [2], [3]. Treatment in primary care may consist of counselling by the general practitioner, various forms of psychotherapy and/or antidepressants [4]. Many studies have provided evidence for continuation of antidepressants after remission to prevent relapses. Far less evidence is available for treatment after this continuation phase, to prevent recurrences, known as maintenance treatment [5], [6]. Most guidelines do recommend maintenance treatment, of various durations, in a subgroup of patients with high risk of recurrence. However, the various guidelines, such as the NICE guideline depression in adults, the ICSI Health Care guideline major depression in adults in primary care and the Dutch General practitioners guideline depression (NHG-standaard Depressieve stoornis) use different indicators for patients at increased risk of recurrence [5], [7]–[11]. Almost all guidelines recommend maintenance treatment with antidepressants in case of recurrent depression, some also after a first episode if it was a severe or chronic episode. Less frequently the following criteria are used in some guidelines: residual symptoms, stressors or lack of support, concurrent other DSM-IV axis I or II disorders, age <30 or >65, rapid relapse or recurrence in the past and family history of major depressive disorder [5].

In a previous paper based on data from the Netherlands Study on Depression and Anxiety (NESDA), we reported that only 5.5% of patients receiving antidepressants in Dutch primary care, do use their antidepressant without a justified indication according to the primary care guidelines depression and anxiety [12]. In the same study we found that over half of the patients without a current justification had started to use antidepressants with a justification in the past. Apparently, a proportion of patients using antidepressants, decide to continue them for years after recovery.

Currently, we do not know which of these patients should indeed be advised to continue using their antidepressant to prevent recurrences and which patients could “safely” be advised to discontinue them. Studying the patients of our previous study in more detail may shed some light on current practice in maintenance antidepressant prescription, which patients or for which patients the decision is made to continue antidepressant medication? More specifically, we were interested to know whether patients using antidepressants as maintenance treatment have ‘valid’ reasons for that according to guideline recommendations. Therefore, we decided to compare sociodemographic, clinical and care characteristics of remitted patients (in remission for at least six months) with and without maintenance treatment (antidepressant use ≥12 months). Subsequently we compared these characteristics with guideline recommendations for maintenance treatment. We hypothesized a priori that most patients on maintenance treatment would meet one or more guideline criteria (Dutch primary care guideline depression 2003) for maintenance treatment such as a recurrent or chronic depression and that these patients more often would have a comorbid anxiety disorder than patients without maintenance treatment.

## Materials and Methods

This study was conducted with data from NESDA (www.nesda.nl), a large prospective cohort study (n = 2981) on the course of depression and anxiety disorders among respondents aged 18–65 years, recruited from the community, primary care and secondary mental health care. Detailed information on the objectives, study population and methods of NESDA has been published [13].

### Study sample

The current study used data from the baseline, two-year and four-year follow-up measurements/interviews of NESDA on only respondents recruited from primary care. We decided to use data on these respondents only since we wanted a representative primary care sample. Recruitment was described in detail elsewhere [13]. Briefly, recruitment in primary care went as follows. A written screener was sent to 23.750 primary care patients that consulted their general practitioner in the past four months, irrespective of the reason for consultation. The screener was returned by 10,706 persons (45%). The non-responders showed no bias with regard to psychopathology [14]. Those screening positive were approached for a telephone interview consisting of the Composite International Diagnostic Interview Short Form sections (CIDI-SF), which has proven diagnostic quality for screening purposes [15], [16]. Respondents fulfilling criteria for a current disorder on the CIDI-SF were invited to participate, as were a random selection of screen-negatives, both from the written screener and the CIDI-SF. In total, 1610 persons were recruited, who underwent an extensive baseline interview, including the CIDI [17], [18]. The GP was not aware of the results of the screening or the interview. After two years and after four years the interview, including the CIDI was repeated.

We included those patients that had recovered from a major depressive disorder at least more than 6 months ago according to the CIDI at that moment (either baseline interview, two-year follow-up or four-year follow-up), i.e. those with a lifetime major depressive disorder but not in the past 6 months (n = 776). Some patients fulfilled the criteria for inclusion on multiple occasions e.g. at baseline and two-year follow-up. We included them separately for each interview moment. In total we had 1571 observations of remitted depression. Not all patients met criteria for remission (>6 months) on all time points. A few patients dropped out after baseline or two-year follow-up, thereby lacking data on subsequent interviews. In most cases not fulfilling criteria for remission was the cause of being not included at that measurement.

### Definition of maintenance treatment and other long-term treatment

All depression guidelines mentioned in the introduction recommend continuation treatment with antidepressants, after having achieved remission with an antidepressant, to prevent relapses. The recommended duration for continuation treatment varies between four and twelve months. Maintenance treatment is defined as all treatment with antidepressants beyond this period. Therefore, in our analysis we defined maintenance treatment as all treatment with antidepressants ≥12 months in patients with depression that had been in remission for at least six months. With short-term use we refer to all use of antidepressants for less than 12 months.

### Determinants of maintenance use

A detailed description of all measures applied in NESDA has been published [13]. All characteristics were measured at each interview.

#### Sociodemographic characteristics

Sociodemographic characteristics including age, gender and education were self-reported by the patient during the interview, work status was assessed with the Trimbos/iMTA questionnaire for assessment of Costs associated with Psychiatric illness [19]–[21].

#### Clinical characteristics

Clinical characteristics including current and past (last 6 months, last year, lifetime) diagnoses of MDD and dysthymia, comorbid anxiety disorders (panic disorder with and without agoraphobia, agoraphobia, social phobia and generalised anxiety disorder) were all assessed with the CIDI and severity of depression with the IDS (Inventory of Depressive Symptomatology) and of anxiety with the BAI (Becks Anxiety Inventory) [22], [23]. The presence of suicide attempts in the past was measured with the Beck Suicide Ideation Scale [24].

Chronic depression, defined as a CIDI diagnosis of depression and symptoms of depression for more than 24 months and recurrent depression defined as more than one episode of MDD in the past, were assessed during the interviews using the CIDI and life chart data. The life chart is a method for recalling depressive or anxious symptomatology, the respondent was asked during the interview to mention several important (personal) events from the last several years and was subsequently asked to recall if there was some depressive (or anxious) symptomatology at that point. The life chart has been proven useful to assess course of illness in patients with mood disorders [25]–[27].

Personality traits (neuroticism and extraversion) were assessed with the Neuroticism-Extraversion-Openness-Five-Factor-Inventory (NEO-FFI). The number of chronic somatic diseases was derived from the Trimbos/iMTA questionnaire for assessment of Costs associated with Psychiatric illness [19]–[21].

#### Care characteristics

During the interviews the respondents were asked if they had had contact with the GP in the last six months, the number of contacts with the GP in the last six months, if any of these contacts with the GP had been about mental problems, the type of help they received (information, a referral to a specialist/mental health care professional, psychotherapy, practical support, skills-training, other help or no help), if they had perceived need for more or any other form of treatment and if they had had contact with primary (social worker, social psychiatric nurse, first line psychologist, psychotherapist) or secondary (psychiatrist, professional from a mental health care organisation) mental health care.

The respondents had been asked to bring all medication they had used in the past month to the interview. The use of antidepressants and benzodiazepines was then recorded by the interviewer according to the World Health Organization Anatomical Therapeutic chemical (ATC) classification. 35.3% of all respondents had forgotten to bring their medication; antidepressant use was based on self-report in these subjects. Use of antidepressants included selective serotonin reuptake inhibitors (ATC-code N06AB), tricyclic antidepressants (N06AA) and other antidepressants (N06AF/N06AX). St. John's wort was not considered an antidepressant. Past use of antidepressants and duration of use of currently used antidepressants was based on self-report.

### Ethical considerations

The study protocol of NESDA was approved centrally by the Ethical Review Board of the VU University Medical Center and subsequently by local review boards of each participating center (Univsersity Medical Center Groningen, Leiden University Medical Center). After full verbal and written information about the study, written informed consent was obtained from all participants at the start of baseline assessment. A full ethics statement of NESDA is found elsewhere.

### Statistical methods

The Statistical Package for the Social Sciences version 20.0 for Mac was used for the descriptive statistics to describe the study population (IBM Statistics, Chicago, USA). The definition “maintenance antidepressant treatment” as described above was used as the dependent variable. We chose to dichotomize this outcome variable (maintenance antidepressant use; n = 271 versus no antidepressant use or acute/continuation antidepressant use n = 1300) since a dichotomous outcome measure simplifies interpretation of the results and enabled us to calculate chances in terms of percentages on patient level in the final prediction model.

The prediction of all independent variables on our dependent variable “maintenance antidepressant treatment” were analysed with bivariate multilevel logistic regression. To prevent multicollinearity, we excluded from these one of each pair of continuous variables with a mutual correlation >0.7 and dichotomous variables with ≤5.0% of respondents in one of the categories.

To determine which variables independently predicted maintenance treatment or other long-term treatment logistic multilevel analysis was conducted using MLwiN 2.25. Multilevel models are hierarchical systems that estimate regression coefficients and their variance components while at the same time correct for the dependency of the repeated measurements (baseline, two-year and four-year follow-up measurements). The first level was defined as observation (within patient), the second level as patient (between patients). The outcome variables represented the logit of the probability (i.e. natural log of the odds) of maintenance antidepressant treatment of depression. Regression coefficients were transformed into odds ratios by taking the EXP[regression coefficient]. The Wald test was used to obtain a p value for each regression coefficient. The Wald test was also used on the variance parameters to obtain an indication of the necessity for allowing a random intercept or regression coefficient into the model [28]. Based on a stepwise backward selection procedure, a final model was fitted consisting of only significant factors that constituted the predictors for long-term/maintenance treatment with antidepressants in the present study.

## Results

### Study sample

The first column of Table 1 lists the characteristics of the study sample. Several dichotomous characteristics had ≤5% in one category and were excluded and not listed in this table (the use of a tricyclic or other antidepressant, whether the respondent had received skills-training, practical support, other help or no help and long-term use of antidepressants in the past).

**Table 1:** Characteristics of all primary care participants and those with remitted depression.

	All measurements (1571)	No antidepressant use (1259)	Acute/continuation antidepressant use (41)	Maintenance antidepressant use (271)
**Sociodemographics**				
Age in years, mean (SD)	48.1 (11.8)	47.5 (12.1)	45.2 (9.6)	51.3 (9.5)
Gender (female)	1136 (72.3%)	912 (72.3%)	30 (73.2%)	195 (72.0%)
Education (high)^1^	700 (44.6%)	598 (47.4%)	16 (39.0%)	88 (32.5%)
Working	1014 (64.5%)	823 (65.3%)	25 (61.0%)	168 (62.0%)
**Clinical characteristics**				
No. chronic somatic diseases, mean (SD)	0.9 (1.1)	0.9 (1.0)	1.0 (1.2)	1.0 (1.2)
IDS^2^ (moderate-very severe)	272 (17.3%)	198 (15.7%)	15 (36.6%)	60 (22.1%)
BAI^3^ (moderate-severe)	174 (11.1%)	126 (10.0%)	8 (19.5%)	40 (14.8%)
Neuroticism, mean (SD)	34.2 (7.7)	33.9 (7.7)	38.1 (7.1)	35.3 (7.8)
Extraversion, mean (SD)	37.6 (6.7)	38.0 (6.5)	35.9 (7.3)	36.0 (7.0)
Suicide-attempt	113 (7.2%)	89 (7.1%)	4 (9.8%)	20 (7.4%)
Dysthymia lifetime	453 (28.8%)	323 (25.6%)	13 (31.7%)	117 (43.2%)
Recurrent MDD	888 (56.5%)	712 (56.5%)	23 (56.1%)	153 (56.5%)
Chronic depression	252 (16.0%)	191 (15.1%)	5 (12.2%)	56 (20.7%)
Anxiety^4^ <12 months	434 (27.6%)	324 (25.7%)	22 (53.7%)	88 (32.5%)
Anxiety^4^ lifetime incl. <6 months	1070 (68.1%)	811 (64.3%)	32 (78.0%)	229 (84.5%)
**Care characteristics**				
Contact with GP <6 months	1232 (78.4%)	982 (77.9%)	34 (82.9%)	217 (80.1%)
No. of contacts GP <6 months, mean (SD)	2.2 (2.5)	2.1 (2.3)	3.3 (3.6)	2.6 (3.4)
Contact GP about mental	219 (13.9%)	140 (11.1%)	24 (58.5%)	56 (20.7%)
Received information	249 (15.8%)	171 (13.6%)	20 (48.8%)	59 (21.8%)
Received referral	199 (12.7%)	128 (10.2%)	21 (51.2%)	51 (18.8%)
Received psychotherapy	346 (22.0%)	252 (20.0%)	24 (58.5%)	71 (26.2%)
Perceived need for more or other treatment	388 (24.7%)	299 (23.7%)	8 (19.5%)	78 (28.8%)
Psychological/psychiatric care past six months^5^	511 (32.5%)	358 (28.4%)	33 (80.5%)	121 (44.6%)
Past antidepressant use	127 (8.1%)	100 (7.9%)	7 (17.1%)	20 (7.4%)
Benzodiazepine use	178 (11.3%)	105 (8.3%)	10 (24.4%)	63 (23.2%)
SSRI^6^ current	248 (15.8%)	N/A	29 (70.7%)	219 (80.8%)

All numbers are number of participants with characteristic (percentage) unless otherwise specified.

In all dichotomous variables 0 = no/characteristic not present, 1 = yes/characteristic present

1 Low-average (elementary (not completed), general intermediate, lower/intermediate vocational, or general secondary education) versus high (higher vocational, college or university education).

2 Inventory of depressive symptomatology; depression severity. None-mild disorder versus moderate to (very) severe disorder.

3 Beck's anxiety inventory; anxiety severity, none-mild disorder versus moderate to severe disorder.

4 Anxiety disorder (panic disorder with or without agoraphobia, agoraphobia, social phobia or generalized anxiety disorder).

5 Primary mental health care/psychological care: social worker, social psychiatric nurse, first line psychologist, psychotherapist; secondary mental health care/psychiatric care: psychiatrist, professional from a mental health care organisation.

6 Selective Serotonin Reuptake Inhibitors.

### Antidepressant and long-term antidepressant use

Out of 1610 primary care respondents, 776 had remitted depression (lifetime MDD and no depression in the past six months), these respondents had a total of 1571 measurements of remitted depression. 1259 times no antidepressant was used, in 41 occasions an antidepressant was currently used for less than 12 months and 271 cases there was maintenance treatment with antidepressants (antidepressant use ≥12 months).

The characteristics of each of these three groups are listed in the right three columns of table 1. As the group of currently acute/continuation users of antidepressants is very small, we compared maintenance users with the “no antidepressant use” and “acute/continuation use” group combined.

### Determinants of maintenance antidepressant use in remitted patients

#### Bivariate analysis

After excluding variables with a mutual correlation >0.7 (received psychotherapy because of correlation with psychological/psychiatric care) and exclusion of the variable current SSRI use (this variable would obscure results as most antidepressants users used an SSRI and almost all antidepressant users were maintenance users), we did a bivariate multilevel logistic regression (table 2). Eight variables were significantly (p<0.05) associated with maintenance treatment with antidepressants.

**Table 2:** Results of bivariate multilevel logistic regression in patients remitted depression* with dependent variable ‘maintenance treatment with antidepressants.’

	Remitted patients (1571 measurements)	
	Odds ratio (95% CI)	p-value
**Sociodemographics**		
Age in years	*1.035 (1.018–1.051)*	*0.000*
Gender (female)	1.016 (0.685–1.507)	0.937
Education^1^ (high)	*1.742 (1.219–2.489)*	*0.002*
Working	0.863 (0.615–1.212)	0.395
**Clinical characteristics**		
No. chronic somatic diseases	1.065 (0.916–1.239)	0.413
IDS^2^ (mod/severe)	1.293 (0.874–1.914)	0.199
BAI^3^ (mod/severe)	1.250 (0.779–2.004)	0.355
Neuroticism	1.015 (0.993–1.037)	0.173
Extraversion	*0.965 (0.940–0.990)*	*0.006*
Suicide attempt	1.024 (0.570–1.841)	0.936
Dysthymia lifetime	*2.226 (1.537–3.223)*	*0.000*
MDD<12 months	1.209 (0.754–1.939)	0.430
Recurrent MDD	1.043 (0.734–1.481)	0.814
Chronic depression	*1.587 (1.017–2.477)*	*0.042*
Anxiety^4^<12 months	1.171 (0.836–1.641)	0.358
Anxiety^4^ lifetime	*2.910 (1.902–4.452)*	*0.000*
**Care characteristics**		
Contact with GP<6 months	1.129 (0.779–1.635)	0.522
No. of contacts GP<6 months	1.045 (0.987–1.106)	0.129
Contact GP about mental problems	1.411 (0.938–2.121)	0.098
Received information	1.406 (0.952–2.077)	0.087
Received referral	1.496 (0.980–2.285)	0.062
Received psychotherapy	1.232 (0.859–1.768)	0.256
Perceived need for more or other treatment	1.174 (0.833–1.654)	0.361
Psychological/psychiatric care^5^	*1.584 (1.149–2.185)*	*0.005*
Past antidepressant use	0.660 (0.364–1.198)	0.172
Benzodiazepine use	*2.389 (1.528–3.735)*	*0.000*

*1571 measurements in 776 individual patients.

In all dichotomous variables 0 = no/characteristic not present, 1 = yes/characteristic present.

1 Low-average (elementary (not completed), general intermediate, lower/intermediate vocational, or general secondary education) versus high (higher vocational, college or university education).

2 Inventory of depressive symptomatology; depression severity. None-mild disorder versus moderate to (very) severe disorder.

3 Beck's anxiety inventory; anxiety severity, none-mild disorder versus moderate to severe disorder.

4 Anxiety disorder (panic disorder with or without agoraphobia, agoraphobia, social phobia or generalized anxiety disorder).

5 Primary mental health care/psychological care: social worker, social psychiatric nurse, first line psychologist, psychotherapist; secondary mental health care/psychiatric care: psychiatrist, professional from a mental health care organisation, care in the past six months.

##### *Sociodemographic characteristics*

Increasing age led to more maintenance treatment, while a high education decreased the chances for maintenance treatment with antidepressants. Personality characteristics were also associated with maintenance treatment with antidepressants. Increasing extraversion led to less maintenance treatment.

##### *Clinical characteristics*

A history of anxiety disorders or dysthymia also led to more maintenance treatment, as did a chronic depression in the past. Recurrent depression was not significant.

##### *Care characteristics*

Receiving care from a mental health professional (psychological or psychiatric care) led to increased chance of maintenance treatment with antidepressants. Finally the use of benzodiazepines increased the ‘risk’ of receiving maintenance treatment with antidepressants. Contact with the GP whether or not about mental problems did not reach significance. Also receiving information or a referral to a specialist remained non-significant.

#### Multivariate analysis

Next, multivariate multilevel logistic regression was performed (table 3). For multivariate analysis, we included all characteristics from the bivariate analyses with p<0.2. Six variables were retained in the final multivariate model. Age (in years), education (0 = low-intermediate, 1 = high), having a history of dysthymic disorder or an anxiety disorder (0 = no, 1 = yes), having received psychological or psychiatric care in the past six months and the current use of benzodiazepines (0 = no, 1 = yes).

**Table 3:** Results of multivariate multilevel logistic regression in patients with remitted depression* with dependent variable “maintenance treatment with antidepressants.”

	Odds ratio	95% CI for odds ratio	p-value
**Sociodemographics**			
Age (in years)	1.033	1.014–1.051	0.000
Education^1^ (high)	0.645	0.440–0.945	0.024
**Clinical characteristics**			
Dysthymia lifetime	1.891	1.290–2.771	0.001
Anxiety lifetime^2^	2.300	1.474–3.589	0.000
**Care characteristics**			
Psychological/psychiatric care past six months^3^	1.644	1.164–2.321	0.005
Benzodiazepine use	2.046	1.283–3.262	0.003

*1571 measurements in 776 individual patients.

In all dichotomous variables 0 = no/characteristic not present, 1 = yes/characteristic present.

1 Low-average (elementary (not completed), general intermediate, lower/intermediate vocational, or general secondary education) versus high (higher vocational, college or university education).

2 Anxiety disorder (panic disorder with or without agoraphobia, agoraphobia, social phobia or generalized anxiety disorder).

3 Primary mental health care/psychological care: social worker, social psychiatric nurse, first line psychologist, psychotherapist; secondary mental health care/psychiatric care: psychiatrist, professional from a mental health care organisation.

## Discussion

### Summary of main findings

Several characteristics of the patient, disease and treatment were associated with maintenance use of antidepressants in remitted depressed patients. Remarkably, both recurrent depression and chronic depression were not, this hypothesis was rejected. Our other hypothesis that patients with a comorbid anxiety disorder would more often be on maintenance treatment with antidepressants was confirmed. A dysthymic disorder in previous history had the same effect, which was unexpected since acute treatment with antidepressants in this disorder is not first step treatment and should be considered as a trial. It could be that GPs view dysthymic disorder as a mild chronic depression, or that these patients are reluctant to stop their antidepressant because of frequent relapses. Older patients and those with a low or intermediate education more often had maintenance treatment with antidepressants. We think that older patients less often ‘ask’ their GP or another physician if a certain medication can be stopped. Patients with a higher education might favour psychotherapy instead of antidepressant treatment, or GPs might think that patients with a lower education are less able to benefit from psychotherapy. The fact that patients on maintenance treatment more often use benzodiazepines is probably related to symptoms of anxiety for which these drugs are frequently prescribed.

Patients on maintenance treatment had received more often psychological/psychiatric care. We expected that this difference was due to the reception of more psychiatric care, since we had expected patients on maintenance treatment to be patients with recurrent, chronic or more severe depression. Therefore we performed a post-hoc analysis and found that patients on maintenance antidepressant treatment had indeed received more psychiatric (19.5% versus 7.3%) and not more psychological (25.0% versus 22.5%) care. These patients could be more severely ill and therefore have a good reason for maintenance antidepressant treatment, or GPs have less insight in patients (previously) treated in secondary mental health care but do repeat their prescriptions as a result of which antidepressant treatment is not critically evaluated.

The number of contacts with the GP and whether the patient had had contact with the GP about mental problems in the last six months were not correlated to maintenance treatment, as we would have expected. An explanation for this could be that patients with a history of depression in general visit their GP frequently and not just those on maintenance treatment with antidepressants.

Severity (IDS) was not significant, probably since severity was not measured at the start of the episode, but instead at predetermined points in time during the interviews, at which time we selected patients in remission, i.e. without current disorder and therefore probably not a high severity score.

### Strengths and limitations

The present study has several strong points. First, our study group was large, especially for a primary care study. Second, the data collected within NESDA is extensive, enabling us to examine many possible determinants. Third, since the GP was unaware of the study diagnosis, all treatment decisions were based on their own judgment, preventing bias. Fourth, since we had several measurements, we could quite accurately determine the time of remission and presence of maintenance antidepressant treatment with antidepressants.

This study also has some limitations that need addressing. First, since variables such as depression and anxiety severity were not measured at the start of the episode or start of the antidepressant, we could not be sure that no relationship between severity and maintenance treatment with antidepressants exists. Next to that, although the CIDI was administered at three different times, we could not be sure of the exact moment of remission and therefore had to use a slightly less accurate definition of maintenance treatment (treatment with antidepressants for ≥12 months, while there was no depression in the past six months), because the guideline recommends continuation treatment for all patients for six months. In addition, we were unable to use GP diagnosis as a predictor, since diagnosis coding was missing in a significant (>25%) percentage of contacts with the GP, therefore we were unable to analyse whether recognition was a significant predictor of (maintenance) antidepressant use. This limitation also meant that diagnosis was solely based on the interview data/the CIDI, it could be that in some cases GP diagnosis was different from the CIDI diagnosis. Finally, duration since last episode was not included in the analyses. And although this was measured in NESDA, we felt that as this was self-reported during the interviews and many patients reported durations exceeding several years, reliability was at best questionable. Reliability of recall of depressive symptomatology has been questioned by several researchers[29], [30]. Therefore we decided not to test this.

### Comparison with literature

Only few articles report on determinants of maintenance treatment with antidepressants in primary care. A few researchers did study risk factors for non-adherence to continuation and maintenance treatment. Burton et al. studied factors associated with the duration of antidepressant treatment, 40% of their patients continued their antidepressant for more than 180 days. They did not find an association between continuation and sociodemographic factors such as age, gender and socioeconomic deprivation. We did find an association between maintenance treatment with antidepressants and both age and education level. Treatment >180 days could be viewed as continuation or maintenance treatment but is probably in most cases shorter than our definition of maintenance treatment. It could be that the differences arise after longer treatment [31]. Holma et al. found several indicators of receiving maintenance treatment in the univariate analyses: number of previous episodes, comorbid somatic disorders and comorbid mental disorders, severity of anxiety, anxiety disorders, positive medication attitude, personality disorder and good adherence during the acute phase of treatment. In their multivariate analysis only good adherence to acute phase antidepressant treatment remained significant, we did not study this, but did find a significant association between maintenance treatment and anxiety disorders as they did in their univariate analyses [32]. Finally Ten Doesschate et al. examined potential predictors of non-adherence to continuation and maintenance antidepressant use and found that in multivariate analysis personality (measured with the Personality Disorder Questionnaire-4+) and a higher education were associated with an increasing likelihood for non-adherence. A higher education decreased likelihood of maintenance treatment in our study, comparable to the result of ten Doesschate et al. [33]. The personality characteristic extraversion was only significant in the bivariate analysis in our study.

We could not find any other studies that had studied or found dysthymia and/or benzodiazepine use to increase likelihood of receiving maintenance treatment with antidepressants.

#### Comparison with guideline recommendations

As mentioned in the introduction, it is also interesting to compare our results with guideline recommendations for maintenance treatment. Depression guidelines, including the Dutch General Practitioners guideline, recommend maintenance treatment with antidepressants for patients at high risk for relapse and/or recurrence or chronic depression. As we stated in a review in 2010, different guidelines have different indicators of patients at high risk for chronic or recurrent course of depression [5]. The Dutch guideline we used, used the following indicators: recurrent or chronic depression and/or failure of non-pharmacological treatment, or in case of residual or recurrent symptoms after phasing out antidepressants [4]. We would expect these established risk factors for unfavourable course to be determinants of maintenance use.

We were very surprised to find that recurrent and chronic depression were not more common in patients with maintenance antidepressant treatment, since these were the two key indications for maintenance antidepressant treatment in patients with depression according to the Dutch General practitioners guideline (and other guidelines). Since chronic depression was significant in the bivariate analysis it could be that any effect was overshadowed in the multivariate analysis by the fact that these patients e.g. more often received psychological or psychiatric care since chronic depression is also an indication for referral [4]. In an article about referral of depressed patients we did indeed find that chronically depressed patients were referred more often [34]. Recurrent depression did not reach significance or even a trend towards significance in the bivariate analysis. We found it difficult to explain this unexpected finding. One explanation would be that maintenance treatment is prescribed more often only to patients with a high number of previous episodes instead of to all patients with a recurrent episode. Since recall bias of number of episodes is a problem, we decided not to analyse number of episodes. The new Dutch GP guideline depression (2012) also recommends reserving maintenance treatment with antidepressants for patients with more than three episodes of depression [35].

The presence of an anxiety disorder increased likelihood of receiving maintenance antidepressant treatment. All anxiety disorders tested in this study are legitimate indications for the prescription of an antidepressant and the guideline anxiety disorders recommends to continue the antidepressant for at least six to twelve months after remission [36]. A significant proportion of our population probably did not use maintenance antidepressant treatment for remitted depression, but instead with a good indication for an anxiety disorder

### Implications for clinical practice and future research

Not only patients with a comorbid anxiety disorder, but also those with a history of a dysthymic disorder, older patients, lower educated patients and those receiving psychiatric care or benzodiazepines more often use maintenance treatment with antidepressants and remarkably not patients with a recurrent or chronic disorder. GPs should be aware of patients with maintenance antidepressant treatment and individually weigh the risks of stopping versus the disadvantages of continuing the drug, together with the patient. As patients with a dysthymic disorder have a questionable indication for antidepressant use, the dubious advantages and more clear disadvantages of continuing should be critically discussed in these patients. In all patients, but maybe especially in older patients and those with a lower education, it might be necessary for the GP to initiate the discussion about continuation or discontinuation of antidepressant treatment, since these patients seem to use maintenance treatment more often while it is unclear if they have a higher risk of recurrence. Finally, in patients referred back from secondary mental health care on antidepressant treatment, the GP might propose a consultation once or twice yearly, as also proposed in the recent new Dutch GP guideline depression. This consultation could according to the new guideline not only be used to discuss the need to continue the antidepressant, but also to notice signs of impending relapse or recurrence at an early stage.

The role of views of the GP has not yet been studied. It would be interesting if a positive or negative attitude of GPs towards both depressed patients, their views of their task in treating depression and their views of the efficacy and place of antidepressants in depression treatment, influences treatment with antidepressants in their patients. It might also be interesting to study cardiovascular risk factors or lifestyle such as smoking habits, body mass index and use of supplements such as fish oil in order to study relation between lifestyle and choice of treatment for MDD. Next to that, additional analysis is needed among antidepressant users to identify those ‘at risk’ for long-term treatment, since in our group also non-users were present. In addition, another interesting group to study in more detail are patients with persisting depression that have been using an antidepressant for over a year. It would be interesting to find out who these, in some way undertreated, patients are and how we could help these patients to recover. Fourth, qualitative studies in patients and GPs would be interesting to shed more light on decision making and reasons behind choices to (dis)continue antidepressants. Finally, it would be very interesting to perform a randomized controlled trial in which patients are either advised to stop or continue an antidepressant to evaluate risk factors for recurrent/chronic depression after (dis)continuation of antidepressants and establish recommendations for maintenance antidepressant treatment based on evidence.
